# Prevalence, Awareness, Treatment and Control of Hypertension in Indonesian Adults Aged ≥40 Years: Findings from the Indonesia Family Life Survey (IFLS)

**DOI:** 10.1371/journal.pone.0160922

**Published:** 2016-08-24

**Authors:** Mohammad Akhtar Hussain, Abdullah Al Mamun, Christopher Reid, Rachel R. Huxley

**Affiliations:** 1 Division of Epidemiology and Biostatistics, School of Public Health, The University of Queensland, Brisbane, Australia; 2 School of Public Health, Curtin University, Perth, Australia; Shanghai Institute of Hypertension, CHINA

## Abstract

**Objective:**

Hypertension is the major driver of the cardiovascular epidemic facing Indonesia in the 21^st^ century. Understanding the socioeconomic inequalities associated with hypertension is essential for designing effective intervention strategies. The aim of the current study was to use sub-nationally representative survey data to examine socio-demographic inequalities in the prevalence, diagnosis and management of hypertension in Indonesian adults.

**Methods:**

We investigated factors associated with hypertension prevalence, diagnosis, treatment and control using data on self-reported diagnosis and treatment, and blood pressure measurements, collected from 9755 respondents aged 40 years and up in the 2007 Indonesian Family Life Survey (IFLS 4).

**Results:**

Age-standardized prevalence of hypertension among the study participants was 47.8% (95% CI: 46.8, 48.9), of which almost 70% were undiagnosed. Hypertension was significantly higher in women than men (52.3% versus 43.1%, p-value<0.001). Prevalence of hypertension increased significantly with ageing (P_*for trend*_ <0.001). Over 91% (men: 92.1%, women: 90.0%) of hypertension cases were uncontrolled. Gender, education and socioeconomic status had differential impact on the diagnosis of hypertension and in receiving treatment.

**Conclusions:**

Overall, less than a third were aware of their hypertension and a quarter of those on medication had their blood pressure effectively controlled. Men and those of younger age were more vulnerable to have undiagnosed and untreated hypertension. Substantial effort should be given to improve awareness about the condition and making provision for early diagnosis and treatment.

## Introduction

Globally, elevated blood pressure is the leading risk factor in terms of its contribution to the burden of cardiovascular disease (CVD) [1,2] with two-thirds of all stroke and half of all coronary heart disease (CHD) attributable to sub-optimal blood pressure levels.[3] In 2000, approximately one billion people worldwide were estimated to be hypertensive, two-thirds of whom resided in lower- and middle-income countries (LMIC) and this figure is predicted to increase to 1.56 billion by 2025 with a disproportionate burden occurring in LMIC.[4]

Intervention studies have clearly demonstrated that blood pressure levels are amenable to intervention through behaviour and lifestyle modification either alone or in conjunction with antihypertensive medication.[1, 4–7] Indeed, a substantial component of the observed declines in CVD rates in high-income countries has been attributable to widespread use of antihypertensive therapy.[8] However, treatment of hypertension is predicated on an individual being aware of their hypertensive status which given the often asymptomatic nature of elevated blood pressure remains a major obstacle–particularly in LMIC, where routine CVD surveillance is limited and access to medical care remains prohibitively expensive for large parts of the population.[9–12] Even in high-income countries, awareness of hypertension remains a challenge with only 50% of the population aware of their hypertensive status decreasing to about 40% in LMIC.[13–16] And of those treated, the proportion of individuals who have their hypertension controlled remains low (25–40%). Moreover, significant socio-economic disparities in the level of awareness, treatment and control of hypertension exist particularly in LMIC.[12]

One of the most populous LMIC is Indonesia with a population of more than 250 million people. Similar to other LMIC the burden of CVD in Indonesia has increased significantly in recent decades: stroke, CHDs and hypertensive heart disease account for more than a third (0.5million) of all deaths in Indonesia with hypertension being one of the leading causes of mortality. [17,18] Although there is some evidence that the mean population level of blood pressure and the prevalence of hypertension has risen in Indonesian adults over past decades, little is known regarding its treatment and control within the population. [17, 19–23] Using national representative data from the fourth wave of the Indonesia Family Life Survey (IFLS-4) conducted in 2007/2008, [24] we report on the prevalence, awareness, treatment and control of hypertension in Indonesian men and women and examine how these patterns differ across social strata.

## Methods

### Study population

Data were sourced from the IFLS-4, a continuing health survey initiated in 1993 with three subsequent rounds of data collection (1997/1998; 2000; 2007/2008) with the fourth wave (IFLS-4) being completed in 2008. [24] The surveys collected information on individual, household and community level data using multistage stratified sampling. The original sampling frame was based on households from 13 out of 27 Indonesian provinces that were selected to maximize the representativeness of the study population and which represented approximately 83% of the Indonesian population in 1993. [25] The sampling and survey methods have been discussed in detail elsewhere. [25] The fourth wave of IFLS included 44,103 (51% women) individuals of all ages—(<1 to 80+ years) from 13,535 households located in both urban and rural areas. [25]

### Ethics approval

This study is based on publicly available de-identified data. The use of the dataset for this study was approved by the School of Public Health Research Ethics Committee, School of Public Health, University of Queensland. The IFLS surveys and their procedures were reviewed and approved by the Institutional Review Boards (IRBs) in the United States (at RAND) and in Indonesia at the University of Gadjah Mada (UGM). Written informed consent was obtained from all respondents prior to data collection.

### Blood pressure and hypertension measurement

Blood pressure was measured thrice at home by specially trained nurses on individuals’ age ≥15 years, using Omron digital self-inflating sphygmomanometers while participants were in a seated position.The first measurement was taken at the beginning of the interview with the subsequent two measures taken during the course of the interview. The average of the three measurements was used for current analysis. Information on awareness, treatment and control of hypertension was based on an interviewer administered questionnaire to all individuals ≥40 years, hence the current analysis is restricted to these individuals.

Individuals with hypertension were defined in one of three ways: i) mean systolic blood pressure (SBP) ≥ 140 mm Hg and/or mean diastolic blood pressure (DBP) ≥90 mm Hg; ii) a self-report diagnosis of hypertension; or iii) self-reported use of blood pressure lowering medication. [26] Respondents were additionally classified as i) pre-hypertensive (SBP 120–139 or DBP80–89 mm Hg); ii) stage 1 hypertensive (SBP 140-159mm Hg or DBP 90–99 mm Hg); or iii) stage 2 (SBP≥160 mm Hg or DBP ≥100 mmHg) hypertensive. [26]

Awareness, treatment and control hypertension were defined using recognised criteria. [10] Awareness of hypertension was defined as self-report of any previous diagnosis of hypertension by a medical professional and was based on the respondents’ response to the following question: *Has a doctor/paramedic/nurse/midwife ever told you that you had hypertension*?” Those answering ‘yes’ were subsequently asked: “*In order to manage your hypertension are you currently taking prescribed medication on a weekly basis*?” Those answering yes to this question were considered to be on treatment. In those reporting to be on antihypertensive medication, control of hypertension was defined as having a mean SBP<140 and DBP <90 mmHg.

### Measurement of body size

Body mass index (BMI<18.5 kg/m^2^: underweight; 18.5–24.9kg/m^2^: normal weight; 25.0–29.9kg/m^2^: overweight and; ≥30.0 kg/m^2^: obese) was derived from height and weight measured during the physical examination. Height was measured with Shorr measuring boards and weight was measured using Seca floor-model scales developed in collaboration with UNICEF. The floor-model scales had a digital read-out and were accurate to the nearest 0.1 kg. Waist measurement was measured using a measuring tape and measurements were recorded to nearest 0.1 cm.

### Socio-demographic characteristics

Socio-demographic information was collected by questionnaire which included questions on area of residence (urban; rural); marital status (currently married or unmarried); highest level of attained education (grouped as illiterate; elementary school; high school (or equivalent) completed; graduate and above); socio-economic development (household wealth index computed based on household assets), health insurance status (non-insured or insured), outpatient health care access (whether visited any outpatient health care clinic in one month prior to survey or not). Principal Component Analysis (PCA) was used to construct a wealth index. [27, 28] Household assets included in the matrix for PCA were house and land occupied by the household, other house/building, land (not used for house or farm), vehicles (cars, boats, bicycles, motorbikes), household appliances (radio, tape recorder, television, fridge, sewing or washing machine, VCD player, mobile phone and others), savings/certificate of deposits/stocks, jewellery and household furniture and utensils; and household characteristics- access to pipe water, both for drinking and other household needs, access to toilet, access to electricity and cooking with electric/gas stove. We assumed that the first principal component is a measure of economic status. [29] The PCA score or the wealth index was categorised into quintiles: Q1(poorest quintile) to Q5(least poor quintile). Sample weights were not used during the PCA operation, but rather when constructing population wealth quintiles. The lifestyle risk factors that were included were current smoking status and level of physical activity. Physical activity was assessed through a set of questions [modified short form of International Physical Activity Questionnaire (IPAQ)] on the types and times of physical activities engaged in, in all parts of life: work, home and exercise. [30] The total duration of activities were transformed to Metabolic Equivalent of Tasks (METs)-minutes and summed to gain an overall estimate of physical activity in a week [http://www.ipaq.ki.se] and further classified as low, moderate and high level of physical activity.[30]

### Statistical analysis

Only respondents with complete information on blood pressure measures and hypertension awareness were included in the analysis. Outcome variables were mean SBP and DBP, prevalence of categories of hypertension and awareness, treatment and control of hypertension. Prevalence of hypertension data were analysed, considering the survey design and standardized using the age and sex distribution of the Indonesia census population in 2010. [31] Logistic regression was used to test for trends across age groups, body mass index (BMI) categories, waist circumference categories, educational level and wealth index quintile with adjustment for age, BMI, education, wealth index, as appropriate, and lifestyle risk factors (smoking and physical activity level). The same procedure was used to estimate adjusted odds ratios (and 95% confidence intervals) for hypertension by level of attained education. Marginal statistics were computed to estimate the predicted probabilities of hypertension across sex and other sociodemographic variables.

Mean levels of SBP and DBP, adjusted for socio-demographic, smoking and physical activity level were estimated in each of the nine ordinal groups of body mass index and eight ordinal groups of waist circumference. Among those with hypertension, the percentages of hypertensive aware of their hypertension, treated, treated and controlled; and aware, but not treated, were estimated in each age group for both sexes as well as for other socio-demographic variables. These percentages (and SEs) were then age standardized with the total sample of hypertensives in this survey used as the standard population. Differences between the groups were tested using χ^2^ test for proportion. Odds ratios for socio-demographic and lifestyle risk factors with uncontrolled hypertension (hypertensives with SB≥140 mm Hg or DBP≥90 mm Hg) by sex were calculated by multiple logistic regression analyses. Stepwise logistic regression analyses were used to evaluate the factors that were independently associated with uncontrolled hypertension in the whole population, and separately in women and men; P-values for covariates to be included in the model were set at 0.05. Likelihood Ratio tests rather than Wald tests were used for deciding which variables to include in the model. Forward and backward stepwise logistic regression analysis produced similar results but only the latter are shown as the model had greater AIC and BIC values compared with the forward model. For all analyses we used inverse probability weights and complete cases were weighted by the inverse of their probability of being a complete case. [32,33] Data were analysed using Stata software version 12.0 for Windows (StataCorp LP, College Station, TX) and for all statistical tests a two-sided p-value < 0.05 was considered statistically significant.

## Results

### Participant characteristics

**Fig 1:**
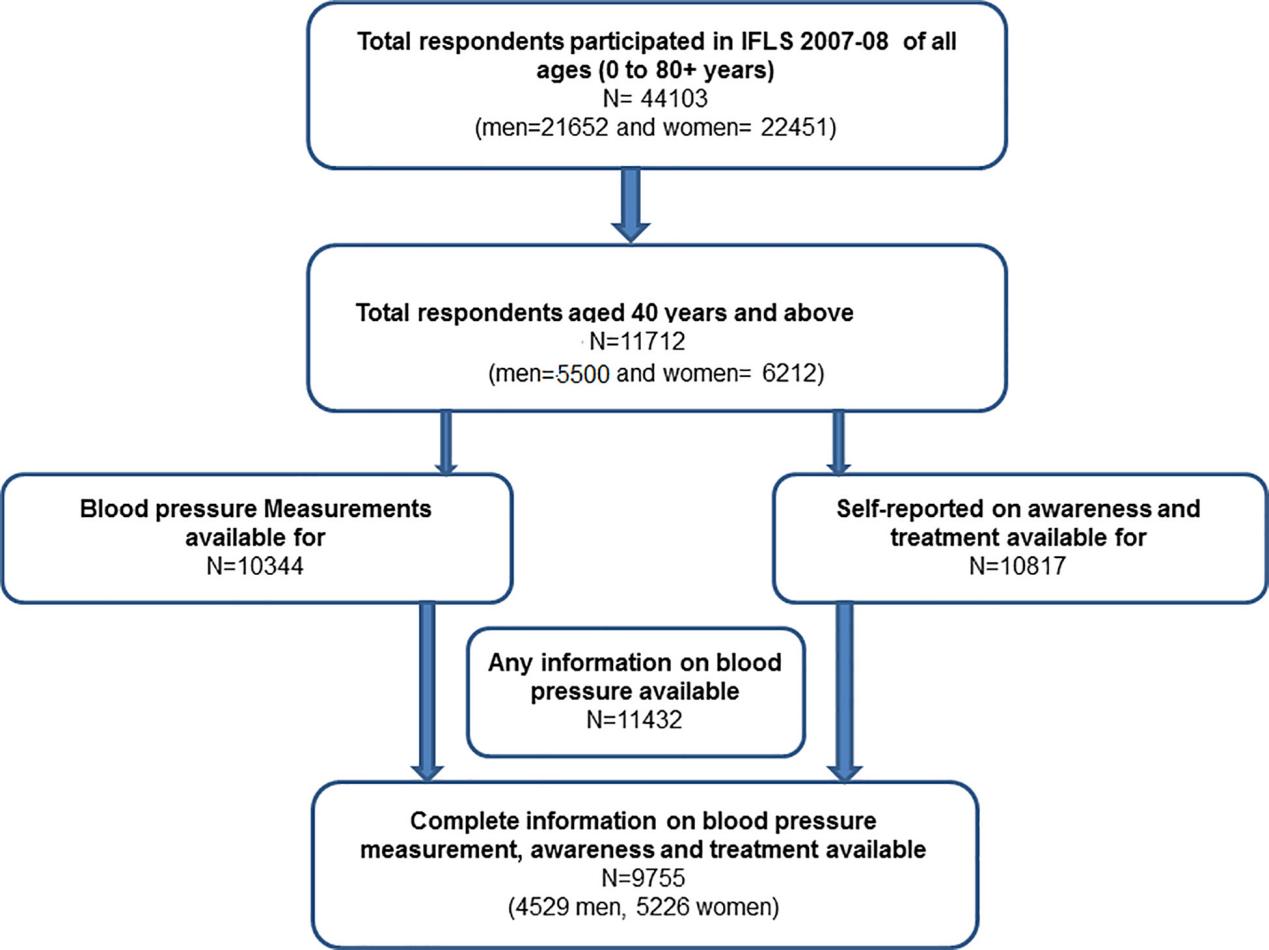
Flowchart showing selection of study sample.

Overall, there were 11712 respondents ≥ 40 years in IFLS-4 (Fig 1). Of these 1677 (14.3%) did not have complete information and were excluded from further analysis, hence the following analysis is based on 9755 participants (85.4%; 53.5% women). Respondents with incomplete information had significantly higher measured SBP compared to those with complete information (152.1 mm Hg vs. 139.5 mm Hg; p<0.001). The median age of the participants was 52 (IQR: 45–62) years and other baseline characteristics are shown in Table 1.

**Table 1:** Baseline characteristics of study population.

	Men		Women		All	
Total	n	Weighted %	n	Weighted %	n	Weighted %
**Age group**	4529	100	5226	100	9755	100
40–49	1903	39.2	2232	42.9	4135	41.1
50–59	1352	33.2	1493	29.4	2845	31.2
60–69	814	18.0	969	18.4	1783	18.2
≥70	460	9.3	532	9.1	992	9.2
**Housing locality**						
Urban	2221	42.4	2653	42.6	4874	42.5
Rural	2308	57.5	2573	57.3	4881	57.4
**Current Marital status**						
Married	4221	93.1	3575	69.2	7786	80.7
Unmarried	318	6.8	1651	30.7	1969	19.2
**Education**						
Illiterate	475	11.2	1307	25.8	1782	18.7
Elementary school	2306	53.3	2634	51.8	4940	52.5
High school	1322	27.1	1029	18.0	2351	22.4
Graduate and above	415	8.2	251	4.3	666	6.2
**Wealth index**						
Q1(poorest quintile)	907	20.2	1180	23.9	2087	22.1
Q2	908	21.3	1022	20.9	1930	21.1
Q3	850	19.7	940	18.5	1790	19.1
Q4	849	18.4	963	17.5	1812	17.9
Q5(least poor quintile)	1016	20.2	1120	18.9	2136	19.5
Health Insurance*						
Yes	1436	29.6	1429	26.3	2865	27.9
No	3093	70.4	3797	73.7	6890	72.1
Health care utilization*						
Yes	627	13.3	1045	19.6	1673	16.6
No	3902	86.7	4180	80.4	8082	83.4
**Body mass index, (kg/m**^**2**^)*						
<18.5	638	15.0	641	13.0	1279	13.8
18.5–24.9	2866	65.9	2547	51.8	5413	58.8
25–29.9	745	15.8	1383	26.3	2128	21.2
≥30	143	3.1	469	8.8	612	6.0
Waist circumference, (cm)*						
<85 for men (<80 for women)	2967	70.5	2164	45.4	5131	57.5
85–94 for men (80–89 for women)	943	19.9	1554	30.5	2497	25.4
≥95 for men (≥90 for women)	441	9.4	1264	24.1	1705	17.0
**Currently smoking**						
Yes	3038	68.6	182	3.4	3220	34.7
No	1419	31.4	5004	96.6	6535	65.2
**Physical activity level**						
Low	868	17.6	1221	22.2	2089	20.1
Moderate	1280	27.6	1957	37.8	3237	32.9
High	2381	54.6	2048	39.9	4429	47.0

### Mean SBP and age-standardized prevalence of hypertension

The overall mean SBP was significantly higher in women (141.0 mmHg; 95% CI, 140.3–141.7) than in men (137.7 mmHg; 95% CI, 137.0–138.2) and rose progressively with age in both sexes (S1 Fig). The age-standardized prevalence of hypertension was 47.8% (Table 2) with 37.4% of the population considered to be ‘pre-hypertensive’ (S1 Table). The prevalence of hypertension was significantly greater in women (52.3% vs. 43.1%; p < 0.001) and in older compared with younger individuals (36.8% in <50 years vs 70.5% in ≥ 70 years). The prevalence of measured hypertension was significantly higher in urban (49.8%) compared with rural (46.4%) areas. In men only, the prevalence of hypertension progressively rose across quintiles of wealth and levels of education (Table 2).

**Table 2:** Prevalence of hypertension by age, sex and socio-economic development in Indonesian men and women, IFLS 2007.

		Men, %(SE)					Women, %(SE)					
	n	40–49 y	50–59 y	60–69 y	≥ 70 y	Total*	40–49 y	50–59 y	60–69 y	≥ 70 y	Total*	Overall population^┼^^a^
**Place of residence**												
Urban	4874	36.7(1.6)	48.2(2.0)	63.0(2.6)	66.4(3.6)	46.2(1.1)	41.4(1.5)	56.2(1.8)	68.4(2.3)	76.7(2.8)	53.3(1.0)	49.8(0.7)
Rural	4881	29.6(1.5)	43.9(2.0)	50.9(2.6)	63.4(3.1)	41.0(1.0)	39.6(1.5)	51.3(2.0)	62.2(2.3)	76.0(2.8)	51.6(1.0)	46.4(0.7)
**Wealth index**												
Q1:poorest quintile	2087	27.9(3.0)	46.2(4.4)	47.9(4.6)	62.6(4.8)	39.7(2.1)	41.5(3.2)	52.6(3.6)	61.8(3.7)	75.4(3.9)	51.2(1.9)	46.4(1.4)
Q2	1930	30.2(2.6)	42.8(3.4)	60.5(4.1)	61.8(5.0)	41.5(1.7)	38.9(2.5)	55.3(3.1)	66.6(3.5)	77.1(4.0)	52.5(1.6)	47.3(1.2)
Q3	1790	32.2(2.2)	41.0(2.8)	56.5(3.7)	66.8(4.9)	41.7(1.5)	41.1(2.1)	49.0(2.8)	65.1(3.5)	77.0(5.2)	51.7(1.4)	46.6(1.0)
Q4	1812	31.8(2.5)	50.8(3.3)	52.9(4.5)	66.7(6.2)	44.0(1.7)	43.2(2.5)	54.8(3.1)	66.5(4.1)	72.2(5.1)	53.7(1.7)	48.9(1.2)
Q5:least poor quintile	2136	39.1(2.5)	50.0(2.9)	59.5(4.3)	66.9(6.2)	48.1(1.7)	37.7(2.2)	57.4(2.9)	63.0(4.0)	81.8(4.5)	52.6(1.5)	50.1(1.1)
**Education**												
Illiterate	1782	28.4(4.7)	42.2(5.1)	61.7(5.0)	59.9(4.4)	40.6(2.8)	38.5(3.2)	49.6(3.1)	62.9(2.6)	74.5(2.7)	49.9(1.7)	46.9(1.5)
Elementary school	4940	31.8(1.6)	44.3(2.0)	51.5(2.5)	67.7(3.2)	41.9(1.0)	43.5(1.5)	53.7(1.8)	67.1(2.5)	78.6(3.3)	54.3(1.0)	48.2(0.7)
High School	2351	32.1(1.9)	48.3(2.6)	60.2(3.9)	64.9(6.5)	44.3(1.4)	35.7(2.1)	56.5(3.2)	62.8(4.4)	83.5(6.7)	51.6(1.6)	47.1(1.1)
Graduate and above	666	40.5(3.5)	52.1(5.0)	64.7(6.9)	51.8(16.5)	48.7(2.8)	35.6(4.0)	60.4(6.1)	54.0(12.4)	1.0	53.2(3.2)	49.4(2.4)
Overall population^a^	9755	32.7(1.1)	45.8(1.4)	55.6(1.9)	64.4(2.4)	43.1(0.7)	40.4(1.1)	53.5(1.4)	64.5(1.6)	76.3(2.0)	52.3(0.7)	47.8(0.5)

* Prevalence standardized for age.

^┼^ Prevalence standardized for age and sex.

^a^ Values for trend by men, *P<0*.*0001*; women, *P<0*.*0001;* social-economic development, *P = 0*.*007;* and education, *P = 0*.*001*.

**Fig 2:**
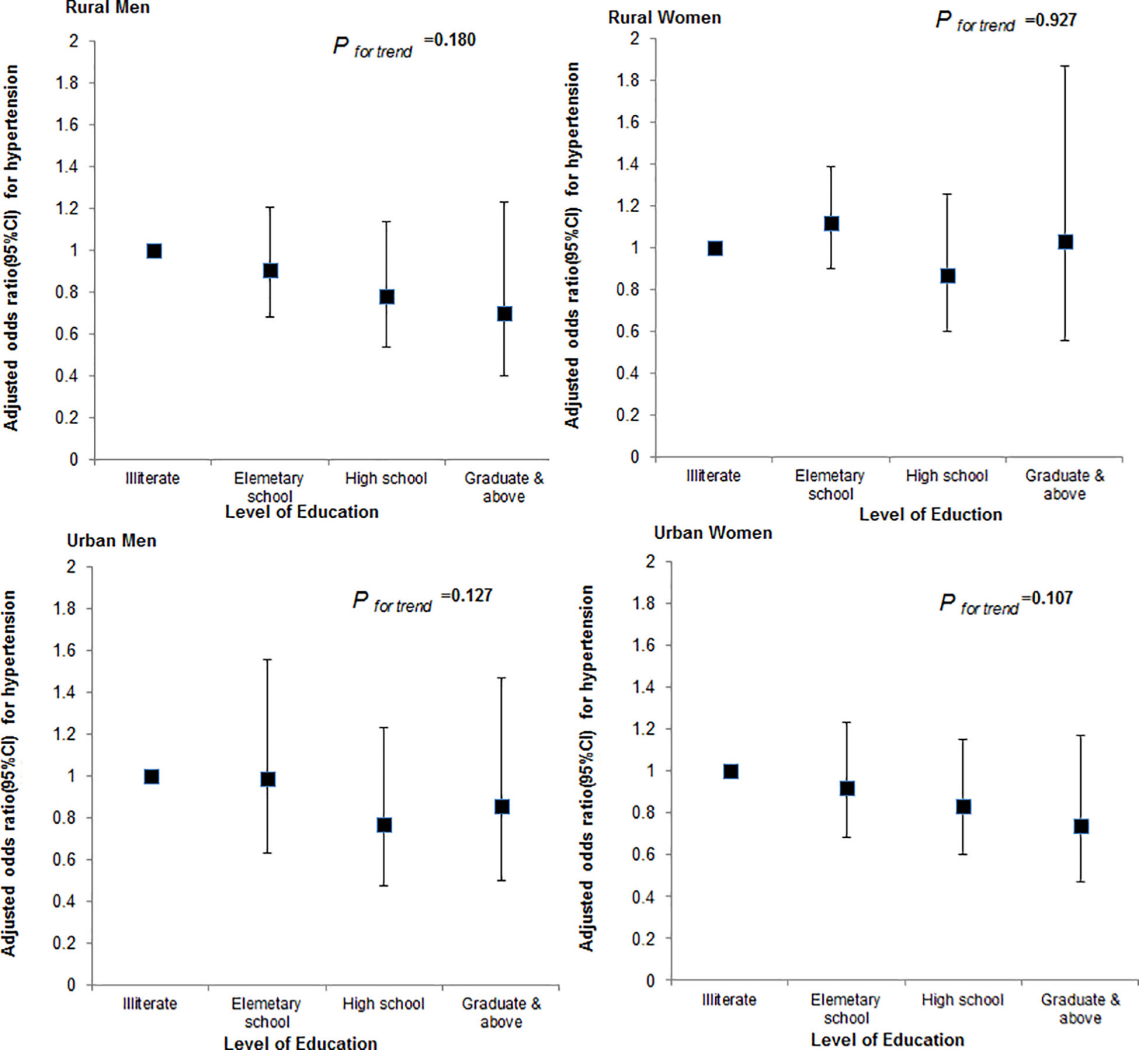
Multiple adjusted odds ratio (AOR) for hypertension (showing 95% CIs) by education status in urban and rural areas in men and women.

**Fig 3:**
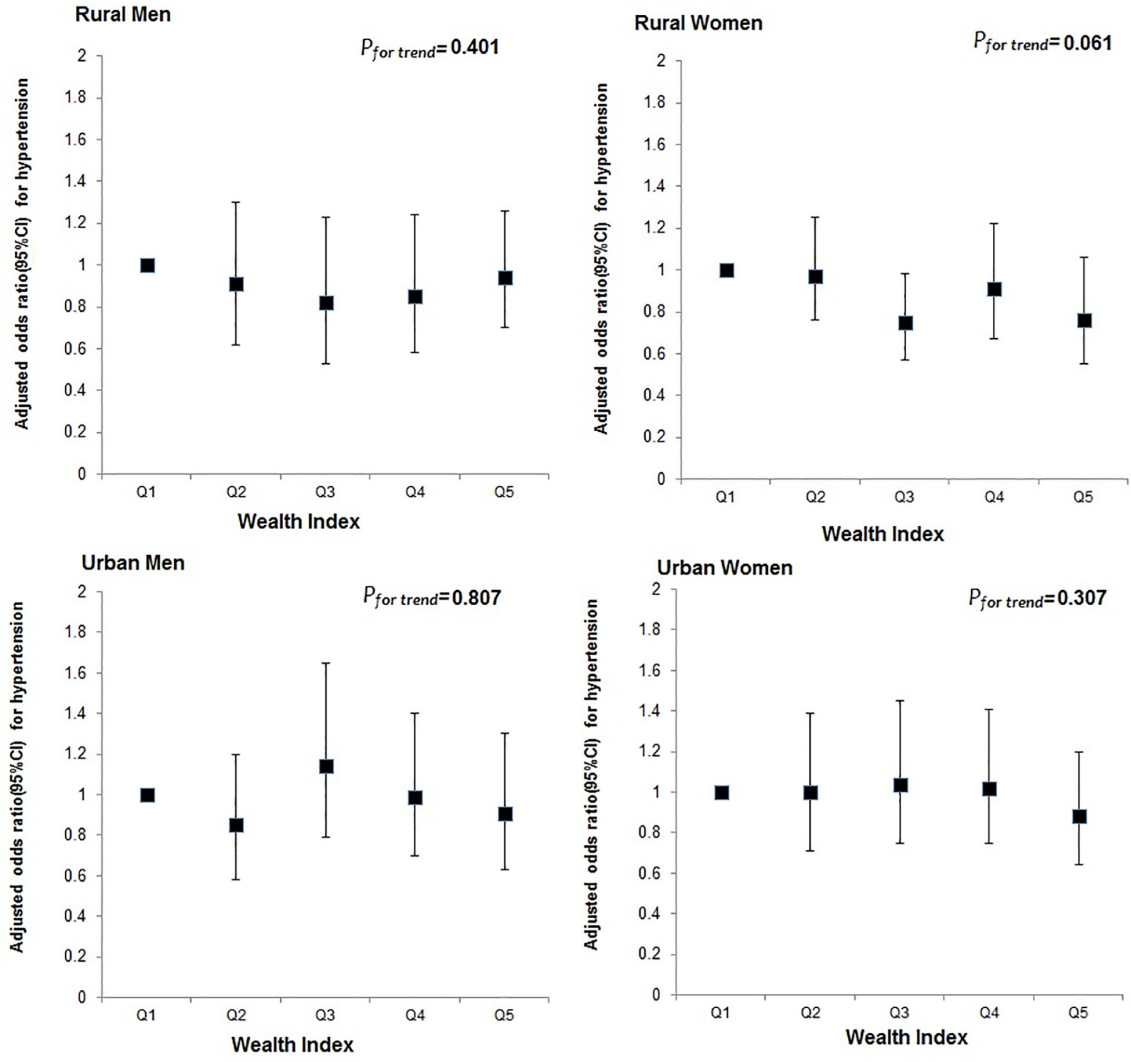
Multiple adjusted odds ratio (AOR) for hypertension (showing 95% CIs) by wealth index in urban and rural areas in men and women.

After adjusting for sociodemographic variables including current smoking and level of physical activity, the odds of hypertension did not vary significantly across level of education (Fig 2) or wealth index (Fig 3) in either men or women.

### Association between body size and blood pressure

**Fig 4:**
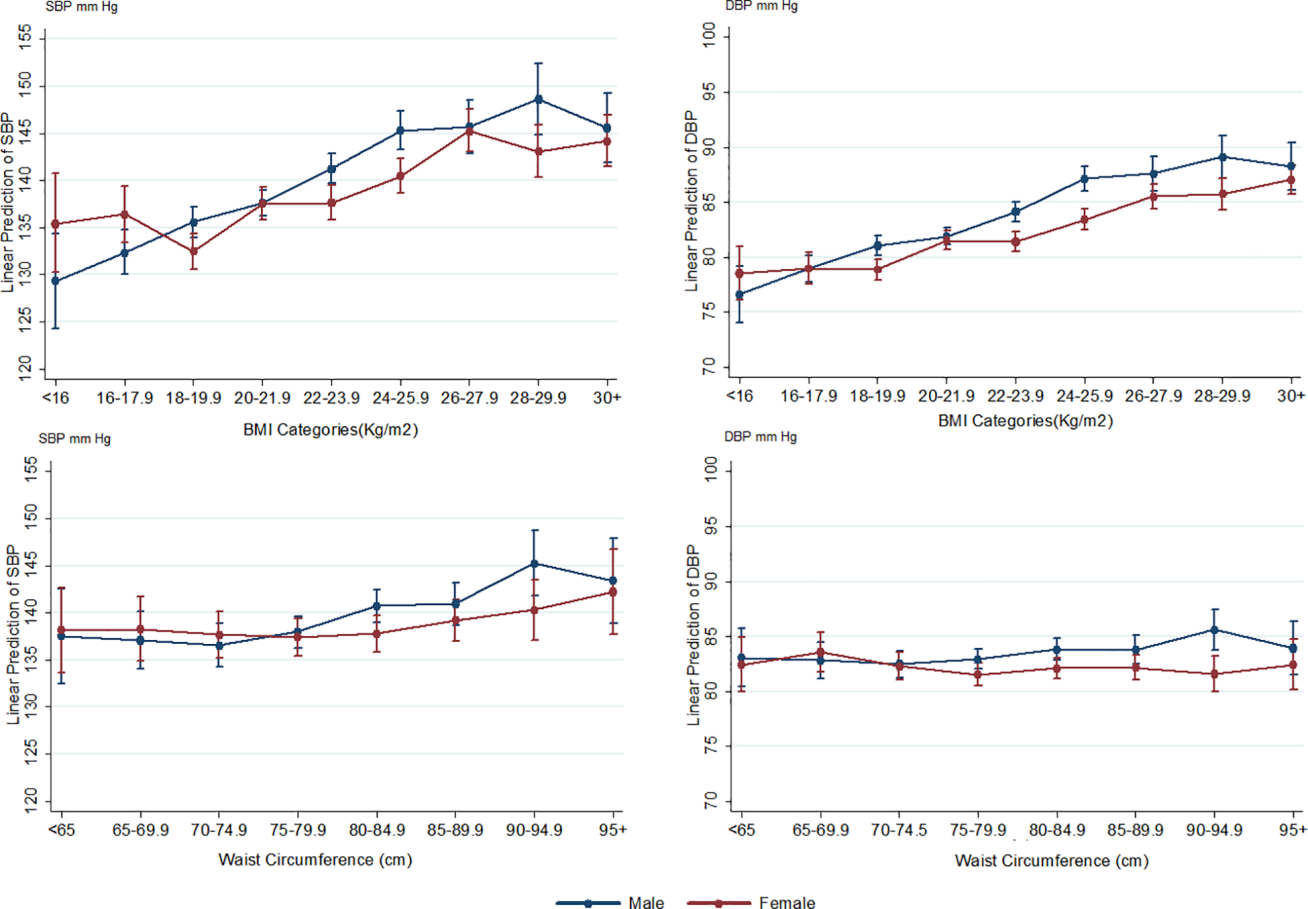
Multiple adjusted mean systolic and diastolic blood pressure (showing 95% CI) across different categories of Body Mass Index and waist circumference in Indonesian men and women ≥ 40 years of age.

Across a wide range of values there was a positive and continuous relationship between BMI and waist circumference with SBP and DBP in both sexes (Fig 4). In men, a one standard deviation increment in BMI and waist circumference was associated with 4.0 mmHg and 3.4 mmHg higher SBP, respectively. In women, the corresponding values were 2.4 mmHg and 2.2 mmHg, respectively. Overall, across BMI and waist circumference (measured on a continuous scale) the adjusted predicted probability of hypertension rose with higher levels of BMI (BMI>30 kg/m^2^) and waist circumference in both men and women (S2 Fig). In men, the odds of being hypertensive increased by 73% (95% CI: 60.3%–87.3%) per one standard deviation increment in BMI and by 70% (95% CI: 58.6% -83.5%) for WC; whereas the corresponding values for women were 55% (95% CI: 44.6%-66.3%) and 47% (95% CI: 37.9%-57.0%), respectively.

### Awareness, treatment and control of hypertension

**Fig 5:**
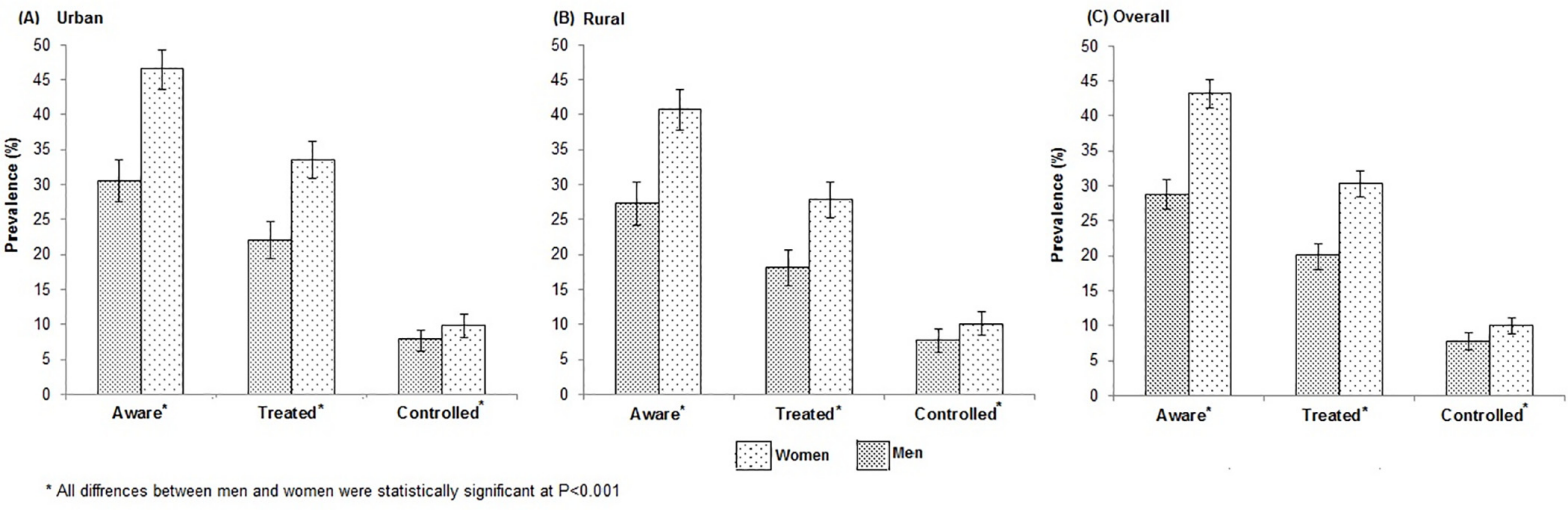
Age-standardized awareness, treated and controlled hypertension prevalence levels (95% CI) in urban (A) and rural (B) areas of Indonesia and (C) overall in men and women with hypertension.

Overall, nearly 37% of Indonesian adults with hypertension were aware of their condition. Significantly more women (43%) were aware of their hypertensive status compared to men (30%; Fig 5) with greater levels of awareness in urban compared with rural areas. Among all hypertensive individuals (irrespective of whether they were aware or not of their condition) about 25% were treated with prescribed antihypertensive medication (which is equivalent to approximately 70% of those who were aware of being hypertensive) with significantly more women than men being treated (Fig 5) irrespective of location. Among those treated, blood pressure control (140/90 mmHg) was achieved in 25% of individuals with slightly better control in men than in women (approximately 27% in men, 24.0% in women) with no significant differences by sex, education, area of residence and wealth index (S2 Table). Overall, adequate control of blood pressure was achieved in about 9% of all individuals with hypertension with similar estimates for rural (9.1%) and urban (9.0%) areas but with greater control occurring in women than in men irrespective of area (Fig 5).

Table 3 shows the results of stepwise logistic regression analysis of associated factors for uncontrolled hypertension in all the respondents, irrespective of sex, and separately for men and women. The factors significantly associated with increased risk of uncontrolled hypertension in overall sample were increasing age and higher waist circumference; whereas, female sex, education, and health care utilisation were associated with significantly lower odds of uncontrolled hypertension. On gender-stratified analysis, elderly age groups were significantly associated with higher odds of uncontrolled hypertension in both women and men (P<0.05). Men and women participants availing health care services (in past one month before survey) had 66% (AOR 0.34; 95% CI: 0.23–0.52) and 47% (AOR 0.53; 95% CI 0.53–0.72) less risk of having uncontrolled hypertension. Overweight and obesity were significantly associated with higher risk of uncontrolled hypertension only in women compared to higher waist circumference only in men. Additionally, education was only a significant factor in women (p<0.05) to be associated with lower odds of uncontrolled hypertension.

**Table 3:** Stepwise logistic regression analysis of factors associated with uncontrolled hypertension.

	Adjusted OR (95% CI)		
	Overall population	Men	Women
**Age groups** (ref:40–49 years)			
60–69 years	2.42(1.72, 3.41)	2.52(1.47, 4.29)	2.05(1.35, 3.10)
70+ years	2.89(1.88, 4.45)	2.22(1.20, 4.11)	3.14(1.83, 5.41)
**Women** (ref. Men)	0.71(0.56, 0.91)	—	—
**Health care utilization** (ref. No)	0.45(0.35, 0.57)	0.34(0.23, 0.52)	0.53(0.39, 0.71)
**Body Mass Index**, kg/m^2^(ref:18–24.9)			
25–29.9	—	—	1.49(1.07, 2.08)
≥30	—	—	1.8(1.16, 2.99)
**Waist Circumference** (ref:<85 for men (<80 for women)			
85–94 for men (80–89 for women)	1.51(1.14, 2.01)	—	—
≥95 for men (≥90 for women)	1.54(1.15, 2.00)	2.00(1.09, 3.66)	—
**Education** (ref: illiterate)			
Elementary school	0.64(0.45, 0.91)	—	0.53(0.35, 0.80)
High school and above	—	—	0.50(0.32, 0.80)

In terms of absolute numbers (as per 2010 census), these estimates would indicate that there are approximately 33 million adults (> 40 years) in Indonesia currently living with hypertension. But of these, only 12 million would be aware of their condition, of whom roughly 8.5 million would be on treatment, but only two million would have their hypertension adequately controlled.

## Discussion

Despite decades of awareness, hypertension remains one of the leading causes of the burden of disease in lower, middle and high-income countries. Findings from this nationally representative study of the Indonesian population illustrate why this remains so. Nearly one-half of all Indonesians aged over 40 years were hypertensive equating to roughly 33 million people–but only 2.5 million of them had their blood pressure adequately controlled with the vast majority of individuals with hypertension living at substantially increased risk of incurring a future vascular event.

Poor blood pressure control is only a part of the explanation for why so few adults with hypertension in Indonesia are effectively treated. In this study, only a third of individuals with hypertension were aware of their condition and although 70% were on treatment, effective blood pressure control was achieved in only a quarter of these individuals. Previously, poor access to medical facilities combined with limited availability of adequate treatments has been reported to be correlated with the level of hypertension in South-Asian populations. Interestingly, among those aware of their hypertension, the ratio of individuals who had their blood pressure controlled to those who were treated was 1:4.3 which is almost identical to that in the Chinese population [9] (1:4.2) whereas in the United States [12] the ratio is 2:3 indicating a much greater effectiveness in controlling blood pressure among those who are treated. Together, these data imply that in Indonesia (and other lower-middle income countries), the most significant obstacles impeding adequate control of hypertension in the population are low awareness (and hence low rates of diagnosis) combined with limited access to effective treatment, and not necessarily treatment initiation among those already diagnosed. Hence, further research on improving blood pressure screening, access to effective medication and treatment adherence strategies in the Indonesian population are warranted.

The prevalence of hypertension reported in this study is notably higher than previous reports from earlier studies that ranged from 5–30%. [21, 24, 35–37] The lower prevalence of hypertension in these surveys could be due to the use of more liberal definitions of hypertension (SBP> = 160 mmHg and DBP> = 95mmHg), [34] and variations in study design, population and methodology. [20, 23, 35] However, reassuringly, the prevalence of hypertension that we report is very similar to the age-specific prevalence reported in cross-sectional national health surveys [20, 36] and similar to that reported from other lower- and middle-income countries. [12]

There were some notable differences in the prevalence of hypertension by sex, age-group and across socioeconomic strata. In agreement with previous findings, the prevalence of hypertension was higher in women than in men [20, 37] but the prevalence of undiagnosed, untreated and uncontrolled hypertension was lower in women compared to men. Of particular concern is that the burden of hypertension was substantially greater in younger versus older individuals; approximately two-thirds of all individuals with hypertension were under 60 years of age. Moreover, the prevalence of undiagnosed and untreated hypertension was also higher in younger (<60 years) compared with older individuals. In contrast to higher-income countries where there is an inverse correlation between the prevalence of hypertension with socio-economic group, in the current study, there was a positive correlation between increasing prevalence of hypertension and increasing affluence(only in men) and increasing level of education in agreement with findings from neighbouring, lower-middle income countries such as Thailand. [38] Compared with urban areas the age-standardized prevalence of hypertension in rural areas was slightly lower (39% versus 35%) whereas individuals living in urban areas were more likely to be treated than those in rural areas, most likely due to greater access to health care. However, once treated, there was no difference between urban and rural areas in the number of individuals who had their hypertension adequately controlled.

One of the factors that may be contributing to the high prevalence of hypertension in the Indonesian population is the increase in the prevalence of overweight and obesity that has occurred in the population possibly as a result of increasing economic development and ‘Westernisation’. [39–41] Recent estimates suggest that one in five Indonesian adult men and one in three adult women are either overweight or obese. [42] In the current study, there was a strong continuous positive association between both BMI and waist circumference (as a marker of central obesity) with blood pressure, which is in agreement with previous reports. [37, 43] Other adverse lifestyle behaviours such as high dietary salt intake and low levels of physical activity which tend to occur with a country’s increasing economic development are also associated with higher blood pressure levels at the population level. Thus, the implementation of effective health promotion campaigns that encourage the maintenance of a normal weight, healthy eating and regular physical activity are likely to have positive effects on mean blood pressure levels in the Indonesian population.

The current study provides important and robust information on levels of awareness, treatment and control of hypertension in the Indonesian population. The strengths of IFLS-4 survey include its high response rate, the representative nature of the study population, and the use of standardized methodology. There are however some important limitations; first, the self-reported information may have been subject to recall bias and may have varied by level of education and an individual’s access to health care. Second, there was no information available for the type of antihypertensive medication that may have been utilised by the study participants, or indeed on duration of usage and adherence to treatment–all of which will impact on blood pressure control. [26] Similarly, we do not have information on whether participants adopted better diet and lifestyle practices as a means of blood pressure control. Finally, we performed a complete case analysis which has the potential to introduce attrition bias as individuals with incomplete data had significantly higher blood pressure levels compared with those with complete data and thereby likely to underestimate the prevalence of hypertension. However, we used inverse probability weighted method to directly analyse only the complete cases with special weights assigned to those cases based on estimated probabilities of completeness, thereby minimising the possibility of attrition bias. [32, 33] Finally, the data on awareness, treatment and control of hypertension was restricted to those aged 40 and over, and therefore we are unable to comment on the burden of hypertension among younger individuals in whom hypertension has been reported to be on the increase. [36]

In summary, more than half of the adult Indonesian population aged over 40 years are hypertensive but only a tiny fraction have their blood pressure adequately controlled. If Indonesia is to achieve the global target of a 25% reduction in the burden of high blood pressure by 2025, [44] then multipronged clinical and public health strategies are urgently required that can i) increase awareness of hypertension in the population, ii) provide better access to more effective blood pressure lowering regimens to a greater proportion of the population and iii) promote population-wide reductions in adverse diet and lifestyle behaviours that are associated with obesity and higher blood pressure levels.

## Supporting Information

S1 Fig Mean level of systolic and diastolic blood pressure (mmHg) in Indonesian men and women. (TIF)

S2 Fig Marginal plot showing predicted probability of hypertension by body mass index and waist circumference in urban and rural areas, and in overall study population (adjusted for age, education, household wealth index, smoking and physical activity level). (TIF)

S1 Table Distribution of measured blood pressure in Indonesian adults ≥ 40 years of age according to various sociodemographic characteristics. (DOCX)

S2 Table Age-standardized percentages of hypertensive, aware, treated, controlled by various socio-demographic characteristics. (DOCX)
