# Supplementary figures and images for: Prevalence, Awareness, Treatment and Control of Hypertension in Indonesian Adults Aged ≥40 Years: Findings from the Indonesia Family Life Survey (IFLS)
Source: PLoS One. 2016 Aug 24;11(8):e0160922. doi: 10.1371/journal.pone.0160922 (PMC4996427; doi:10.1371/journal.pone.0160922)

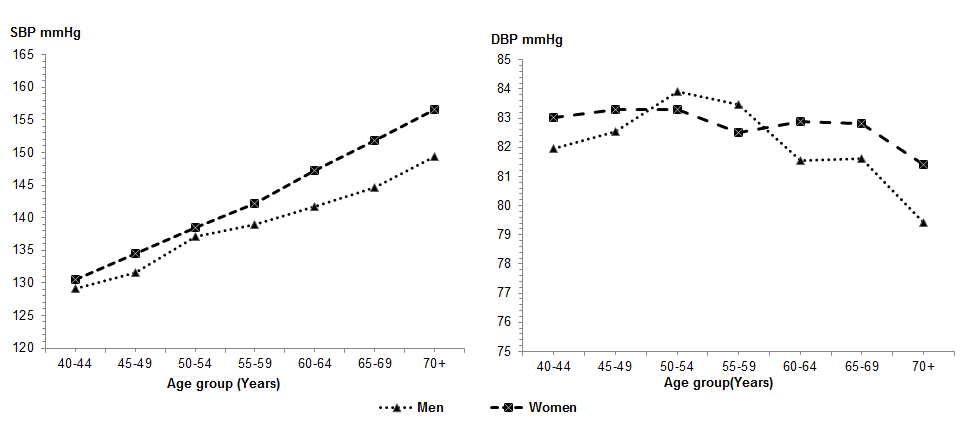

Supplement: S1 Fig — (TIF) [file pone.0160922.s001.tif]

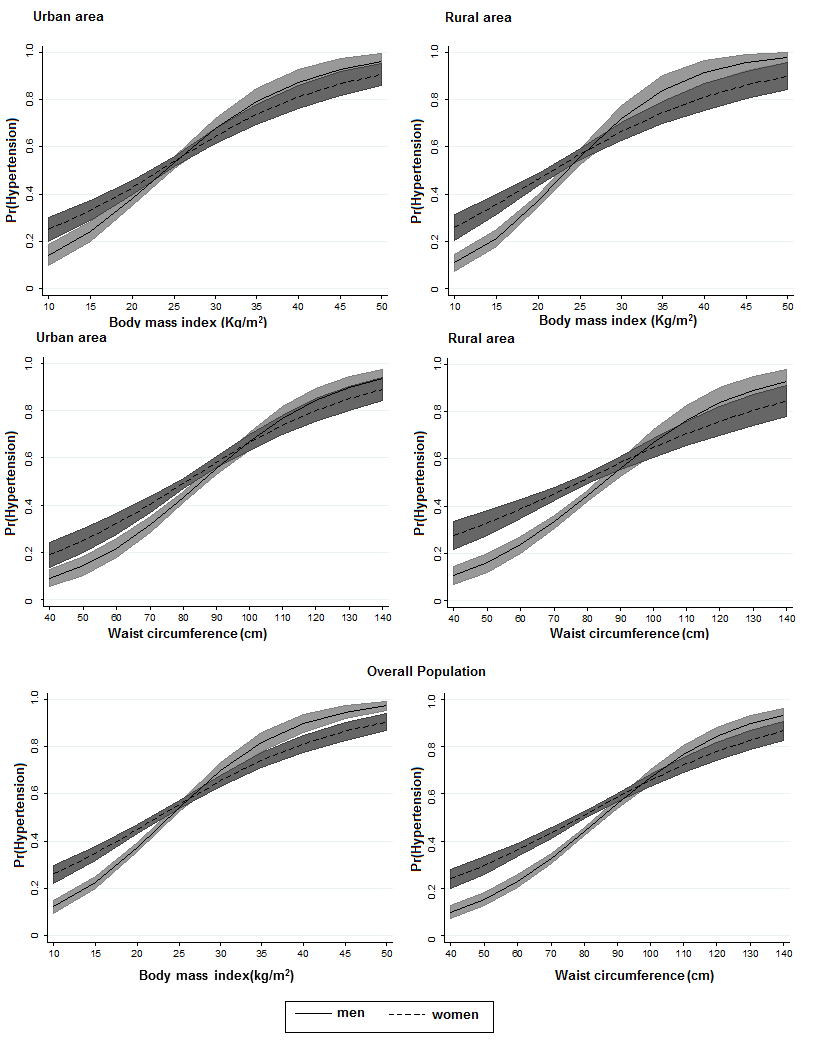

Supplement: S2 Fig — (TIF) [file pone.0160922.s002.tif]
