# Supplementary material for: Prevalence, Awareness, Treatment and Control of Hypertension in Indonesian Adults Aged ≥40 Years: Findings from the Indonesia Family Life Survey (IFLS)
Source: PLoS One. 2016 Aug 24;11(8):e0160922. doi: 10.1371/journal.pone.0160922 (PMC4996427; doi:10.1371/journal.pone.0160922)
Supplement: S1 Table — (DOCX) [file pone.0160922.s003.docx]

**S1 Table-** Distribution of measured blood pressure in Indonesian adults ≥ 40 years of age according to various sociodemographic characteristics.

|  |  | **Normal** | | | **Prehypertension** | | | **Hypertension**  **(Stage 1)** | | **Hypertension (Stage 2)** | |  |
| --- | --- | --- | --- | --- | --- | --- | --- | --- | --- | --- | --- | --- |
|  | **Total** | **n** | | **% (SE)** | **n** | | **% (SE)** | **n** | **% (SE)** | **n** | **% (SE)** | |
| **All** | 10344 | 1847 | | 17.7(0.4) | 3798 | | 37.4(0.5) | 2545 | 24.7(0.4) | 2154 | 20.1(0.4) | |
| **Sex*** |  |  | |  |  | |  |  |  |  |  | |
| Men | 4760 | 837 | | 17.2 (0.5) | 1944 | | 41.6(0.7) | 1174 | 24.7(0.6) | 805 | 16.3(0.5) | |
| Women | 5584 | 1010 | | 18.0(0.5) | 1854 | | 33.6(0.6) | 1371 | 24.7(0.6) | 1349 | 23.5(0.5) | |
| p-value |  | 0.653 | | | <0.001 | | | 1.0 | | <0.001 | |  |
| **Age** |  |  | |  |  | |  |  |  |  |  | |
| 40-49 | 4198 | 1039 | | 24.3(0.7) | 1795 | | 43.3(0.8) | 879 | 20.9(0.6) | 485 | 11.4(0.5) | |
| 50-59 | 2904 | 491 | | 16.5(0.7) | 1099 | | 38.5(0.9) | 723 | 25.3(0.8) | 591 | 19.5(0.7) | |
| 60-69 | 1901 | 219 | | 11.6(0.8) | 572 | | 31.0(1.1) | 553 | 28.8(1.1) | 557 | 28.4(1.1) | |
| 70+ | 1341 | 98 | | 7.3(0.7) | 332 | | 25.0(1.2) | 390 | 29.8(1.3) | 521 | 37.9(1.4) | |
| p-value for trend |  | <0.001 | | | <0.001 | | | <0.001 | | <0.001 | |  |
| **Place of residence** |  |  | |  |  | |  |  |  |  |  | |
| Urban | 5181 | 881 | | 17.6(0.5) | 1850 | | 36.1(0.7) | 1301 | 25.1(0.6) | 1149 | 21.6(0.5) | |
| Rural | 5163 | 1049 | | 18.1(0.5) | 2013 | | 38.3(0.7) | 1245 | 24.4(0.6) | 1007 | 19.0(0.5) | |
| p-value |  | 0.775 | | | 0.157 | | | 0.682 | | 0.134 | |  |
| **Education** |  |  | |  |  | |  |  |  |  |  | |
| Illiterate | 1785 | 247 | | 13.9(0.9) | 588 | | 33.8(1.2) | 485 | 27.4(1.1) | 465 | 24.7(1.1) | |
| Elementary school | 4948 | 941 | | 18.5(0.5) | 1861 | | 38.5(0.7) | 1173 | 23.9(0.6) | 973 | 18.9(0.5) | |
| High School | 2356 | 467 | | 20.3(0.8) | 934 | | 39.8(1.0) | 562 | 23.4(0.9) | 393 | 16.3(0.8) | |
| Graduate and above | 667 | 126 | | 18.4(1.6) | 259 | | 39.1(2.0) | 172 | 25.9(1.8) | 110 | 16.4(1.5) | |
| p-value for trend |  | 0.107 | | | 0.052 | | | 0.398 | | 0.002 | |  |
| **Wealth Index** | |  | |  |  | |  |  |  |  |  | |
| Q1 | 2231 | 360 | | 15.7 (0.8) | 789 | | 35.4(1.1) | 541 | 24.8(1.0) | 542 | 23.9(0.9) | |
| Q2 | 2040 | 382 | | 18.5(0.9) | 759 | | 38.0(1.1) | 488 | 23.6(1.0) | 411 | 19.6(0.9) | |
| Q3 | 1897 | 360 | | 19.1(0.9) | 714 | | 38.6(1.2) | 466 | 24.5(1.0) | 357 | 17.6(0.9) | |
| Q4 | 1895 | 317 | | 16.6(0.9) | 714 | | 38.5(1.2) | 486 | 25.8(1.1) | 378 | 18.8(0.9) | |
| Q5 | 2271 | 423 | | 18.0(0.8) | 821 | | 36.8(1.1) | 563 | 25.2(1.0) | 464 | 20.3(0.9) | |
| p-value for trend |  | 0.636 | | | 0.563 | | | 0.622 | | 0.095 | |  |
| **Currently smoking** | |  |  | |  |  | |  |  |  |  |  |
| Yes | 3223 | 644 | 19.7(0.07) | | 1355 | 42.7(0.09) | | 755 | 23.3(0.08) | 469 | 14.2(0.06) |  |
| No | 6545 | 1138 | 17.1(0.05) | | 2296 | 35.5(0.06) | | 1638 | 25.2(0.05) | 1473 | 21.9(0.05) |  |
| p-value |  | 0.17 | | | 0.000 | | | 0.315 | | <0.001 | |  |

**Normal:** SBP <120 and DBP < 80; **Prehypertension**: 120 ≤ SBP <140 or 80≤ DBP <90; **Stage 1**: 140 ≤ SBP < 160 or 90 ≤ DBP<100; **Stage 2**: SBP ≥160 or DBP ≥ 100; regardless of medication use.
