# Supplementary material for: Prevalence, Awareness, Treatment and Control of Hypertension in Indonesian Adults Aged ≥40 Years: Findings from the Indonesia Family Life Survey (IFLS)
Source: PLoS One. 2016 Aug 24;11(8):e0160922. doi: 10.1371/journal.pone.0160922 (PMC4996427; doi:10.1371/journal.pone.0160922)
Supplement: S2 Table — (DOCX) [file pone.0160922.s004.docx]

**S2 Table**- Age-standardized percentages of hypertensive, aware, treated, controlled by various socio-demographic characteristics.

|  | **Aware** | **Treated** | **Treated and controlled** | **Aware, but not treated** |
| --- | --- | --- | --- | --- |
|  | **% (SE)** | **% (SE)** | **% (SE)** | **% (SE)** |
| **Sex** |  |  |  |  |
| Men | 28.7(1.0) | 19.9(0.9) | 26.9(2.5) | 31.4(2.2) |
| Women | 43.1(1.0) | 30.2(0.9) | 24.0(1.6) | 29.8(1.4) |
| p-value | <0.001 | <0.001 | 0.471 | 0.862 |
| **Age** |  |  |  |  |
| 40-49 | 35.7(1.3) | 22.9(1.1) | 32.6(2.6) | 35.7(2.1) |
| 50-59 | 38.9(1.3) | 27.3(1.2) | 22.8(2.1) | 29.6(2.1) |
| 60-69 | 35.7(1.5) | 27.3(1.4) | 15.6(2.2) | 23.4(2.2) |
| 70+ | 37.9(1.6) | 29.6(1.4) | 13.7(2.5) | 21.9(2.8) |
| P _for trend_ | 0.639 | <0.001 | <0.001 | <0.001 |
| **Housing locality** |  |  |  |  |
| Urban | 39.2(1.0) | 28.3(0.9) | 23.9(1.8) | 28.1(1.6) |
| Rural | 35.0(0.1) | 23.6(0.9) | 25.1(2.0) | 32.4(1.8) |
| p-value | <0.001 | <0.001 | 0.346 | 0.127 |
| **Marital status** |  |  |  |  |
| Currently married | 36.8(0.8) | 25.8(0.7) | 25.1(1.5) | 29.9(1.3) |
| Currently unmarried | 35.4(1.9) | 24.3(1.6) | 26.8(4.2) | 31.4(3.4) |
| p-value | 0.280 |  |  | 0.261 |
| **Education** | |  |  |  |
| Illiterate | 30.5(2.1) | 20.4(1.7) | 21.1(4.3) | 33.2(4.0) |
| Elementary school | 39.4(1.0) | 27.5(0.9) | 26.3(1.8) | 30.1(1.6) |
| High school | 38.2(1.6) | 27.4(1.4) | 22.9(2.5) | 28.9(2.4) |
| Graduate and above | 37.3(3.1) | 25.9(3.0) | 16.8(4.6) | 31.8(5.0) |
| P _for trend_ | 0.006 | 0.064 | 0.597 | 0.597 |
| **Wealth index** |  |  |  |  |
| Q1(poorest quintile) | 29.0(2.0) | 17.8(1.6) | 26.2(4.4) | 38.2(3.9) |
| Q2 | 34.1(1.7) | 24.3(1.5) | 25.1(3.3) | 28.6(2.8) |
| Q3 | 37.7(1.5) | 26.7(1.3) | 25.9(2.6) | 29.0(2.3) |
| Q4 | 41.1(1.7) | 29.1(1.6) | 23.8(2.8) | 28.9(2.5) |
| Q5(least poor quintile) | 41.1(1.6) | 28.9(1.4) | 24.1(2.8) | 30.5(2.5) |
| P _for trend_ | <0.001 | <0.001 | 0.816 | 0.259 |
| Overall | 36.9(0.7) | 25.7(0.6) | 24.7(1.3) | 30.3(1.2) |
